# 3-D twelve-port multi-service diversity antenna for automotive communications

**DOI:** 10.1038/s41598-021-04318-0

**Published:** 2022-01-10

**Authors:** Lekha Kannappan, Sandeep Kumar Palaniswamy, Malathi Kanagasabai, Preetam Kumar, M. Gulam Nabi Alsath, Sachin Kumar, Thipparaju Rama Rao, Mohamed Marey, Apeksha Aggarwal, Jayaram K. Pakkathillam

**Affiliations:** 1 Department of Electronics and Communication Engineering, SRM Institute of Science and Technology, Kattankulathur, 603203 India; 2 Department of Electronics and Communication Engineering, College of Engineering, Guindy, Anna University, Chennai, 600025 India; 3 Department of Electrical Engineering, Indian Institute of Technology, Patna, 801106 India; 4 Department of Electronics and Communication Engineering, Sri Sivasubramania Nadar (SSN) College of Engineering, Chennai, 603110 India; 5 Smart Systems Engineering Laboratory, College of Engineering, Prince Sultan University, Riyadh, 11586 Saudi Arabia; 6 Department of Computer Science Engineering & Information Technology, Jaypee Institute of Information Technology, Noida, 201309 India; 7 Department of Electronics and Communication Engineering, Amrita School of Engineering, Amrita Vishwa Vidyapeetham, Chennai, 601103 India

**Keywords:** Engineering, Physics

## Abstract

This paper presents a twelve-port ultra-wideband multiple-input-multiple-output (MIMO)/diversity antenna integrated with GSM and Bluetooth bands. The twelve-port antenna is constructed by arranging four elements in the horizontal plane and eight elements in the vertical plane. The antenna element, which is created using a simple rectangular monopole, exhibits a frequency range of 3.1 to 12 GHz. The additional Bluetooth and GSM bands are achieved by introducing stubs into the ground plane. The size of the MIMO antenna is 100 × 100 mm^2^. The antenna offers polarization diversity, with vertical and horizontal polarization in each plane. The diversity antenna has a bandwidth of 1.7–1.9 GHz, 2.35–2.55 GHz, and 3–12 GHz, the radiation efficiency of 90%, and peak gain of 2.19 dBi. The proposed antenna offers an envelope correlation coefficient of < 0.12, apparent diversity gain of > 9.9 dB, effective diversity gain of > 8.9 dB, mean effective gain of < 1 dB, and channel capacity loss of < 0.35 bits/s/Hz. Also, the MIMO antenna is tested for housing effects in order to determine its suitability for automotive applications.

## Introduction

In today's fast-paced world, there is an increasing demand for connected vehicles, which allow automobiles to communicate with one another. Vehicles could be linked to more communication devices in the future to provide a more comprehensive, autonomous, and intelligent driving experience. This necessitates the use of automotive antennas capable of supporting multiple frequency bands/vehicular wireless services. However, multiple antennas increase the complexity of the transceiver and also require a large space for their integration on the printed circuit board^1^. A multiband antenna, on the other hand, can be designed to combine multiple frequencies into a single antenna and may serve as the foundation for future development in automotive applications.

Automotive multiband antennas are required for a variety of applications in intelligent transportation systems (ITS), such as vehicle-to-vehicle (V2V), vehicle-to-infrastructure (V2I), and vehicle-to-everything (V2E) communication. The automotive antenna may play a role in the advanced driver assistance system (ADAS), which is a collection of active safety systems that allow drivers to take timely control of their vehicles by warning them of potential road hazards. In the context of automobiles, the ADAS system includes autonomous parking, congestion avoidance via re-routing, and blind spot detection. The Internet of things (IoT) facilitates this ADAS system. The term “automotive IoT” refers to the incorporation of IoT technologies into automotive systems in order to develop new applications and solutions that can make vehicles smarter and more intelligent, resulting in safer, more efficient, and more comfortable driving. Vehicle IoT technology enables applications such as autonomous driving, braking, automatic parking, traffic tracking, route and driver control.

Recently, a few ultra-wideband (UWB) antennas with integrated multi-standard bands have been reported for automotive applications. Despite the numerous advantages of UWB technology, multipath propagation and fading degrade system performance by decreasing the signal to interference ratio. The fading problem can be alleviated by introducing a diversity scheme. Diversity improves signal reliability by obtaining replicas of the information signal across multiple pathways. The combination of multiple-input-multiple-output (MIMO) and UWB technologies can improve system robustness by avoiding the effects of fading and multipath propagation. MIMO transmits and receives uncorrelated signals while increasing channel capacity by forming parallel resolvable channels. However, the main challenges in MIMO antenna design are high inter-element coupling and compact size suitable for integration with other high-frequency devices^2^. In^3^, a UWB antenna with GSM, WCDMA, and WLAN integrated bands was presented. The ground plane of the antenna was modified with capacitively loaded line resonators. The multiband operation was achieved without increasing the size of the antenna, but the antenna showed single polarization. In^4^, a rectangular patch antenna with multiple standards was reported, where an octagonal-shaped slot was used to integrate multiple bands. In^5^, slots were introduced in the ground plane to achieve multiple band resonance without increasing the physical size of the antenna. In^6^, a compact UWB monopole antenna with a notch and resonating strips was designed to achieve the quad-band performance. In^7^, a compact-sized UWB antenna with band-notched characteristics was developed. The antenna offered good isolation, but its polarization was limited. In^8^, a dual-polarized UWB MIMO antenna with integrated 1.9 GHz and 2.4 GHz was presented. In^9^, a MIMO antenna was designed with good isolation for IEEE 802.11 a/b/g/n applications, however, only single polarization was obtained. The band-notched multiband antennas were designed in^10–12^. In^13^, a UWB MIMO antenna with improved isolation and dual polarization was proposed. In^14^, a quad-port UWB antenna with an integrated GSM band was proposed without increasing the overall antenna size. The antenna offered horizontal and vertical polarization. In^15^, a uniplanar four-port differently driven UWB antenna was presented, where high isolation and low cross-polarization were achieved through different feeding mechanisms. In^16^, a UWB antenna integrated with Bluetooth and WLAN bands was presented, where ring slots were loaded in the patch for achieving multiband characteristics. However, the overall size of the antenna element was larger. In^17^, the antenna elements were located perpendicular to each other, and good isolation was obtained without any isolation technique. In^18^, an RF amplifier was integrated with the UWB MIMO antenna, but only one type of polarization was achieved. In^19^, a compact broadband MIMO antenna for indoor wireless communication systems was proposed. The antenna offered good isolation without the use of decoupling structures, but it was limited to two polarization vectors. In^20^, eight differentially-fed microstrip antenna elements with dual polarization were arranged. The antenna covered the N79 band for 5G, but it had a low efficiency. In^21^, a slit/slot antenna fed by a transmission line was proposed for tri-polarized MIMO applications. A tri-polarized single-layer MIMO antenna with vias, which allows the different modes to resonate at the same frequency, was reported in^22^. However, the antenna geometry in the majority of the above-mentioned designs was complex and difficult to integrate with other circuits.

In this paper, a MIMO antenna with twelve resonators arranged in horizontal and vertical planes is proposed. The main features of the presented work are:

1. The antenna covers two narrow bands (GSM and Bluetooth) and the entire UWB. Numerous wireless services required in automobiles are integrated into a single radiator, eliminating the need for multiple patches.
2. The 3-D orientation of the radiators reduces the total area occupied by the antenna, allowing more elements to be incorporated into a small space.
3. The polarization diversity is achieved by arranging the radiators orthogonally to each other.
4. Placing the antenna elements in both the E-plane and the H-plane result in additional polarization. In comparison to other antennas in the literature, the proposed design generates additional polarization vectors, resulting in a more robust diversity scheme.
5. The link reliability and channel capacity are improved due to the increased degree of freedom offered by the proposed antenna.
6. Isolation greater than 20 dB is obtained, without the usage of any decoupling structures.
7. The housing effects are investigated for the reliability test of the antenna for automotive applications. The horizontal and vertical orientations of the proposed antenna are tested in the presence of conducting bodies. The housing effects results validated the stability of the antenna.
8. The far-field performance of the proposed antenna on the vehicle is investigated, and the results show that the antenna exhibits omnidirectional characteristics when placed on the car body.

First and second sections present the design of the antenna element and MIMO antenna, respectively. Third section presents the results and diversity characteristics of the antenna. The antenna housing effects are discussed in fourth section, and fifth section presents the conclusion.

## Antenna design

### Evolution of the UWB antenna element

**Figure 1:**
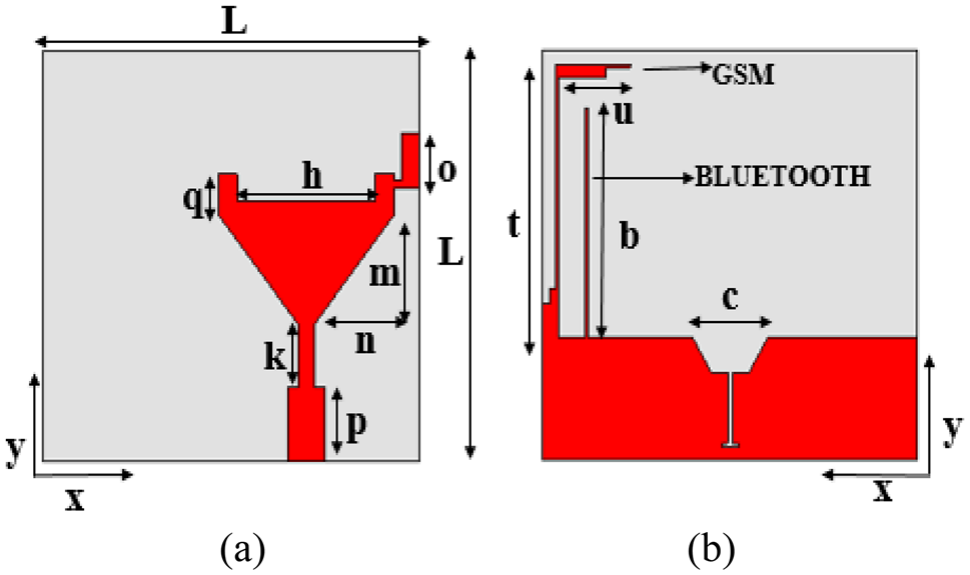
Proposed antenna element: (**a**) front view and (**b**) back view.

The proposed UWB monopole antenna element is depicted in Fig. 1. The overall size of the antenna element is 30 × 30 mm^2^. The antenna element is designed on the FR-4 substrate with relative permittivity of 4.4, loss tangent of 0.025, and thickness of 1.6 mm. The design equation for the UWB planar monopole antenna is given as^23,24^

1 $$f_{l} = \frac{7.2}{{\left( {l + r + p} \right) \times k}}$$

where $f_{l}$ is the lowest resonating frequency of the antenna and *p* is the distance between the patch and the ground plane, and the empirical constant *k* is calculated as

2 $$k = \sqrt[4]{{\varepsilon_{eff} }}$$

For the proposed antenna, Eq. (1) is modified as

3 $$f_{l} = \frac{7.2}{{\left( {0.335\pi \left[ {\left( {a + b} \right)} \right] + p} \right) \times k}}$$

where $0.335\pi [(a + b)]$ corresponds to the expression (l + *r*), and the semi-length and semi-width are denoted by *a* and *b*, respectively.

**Figure 2:**
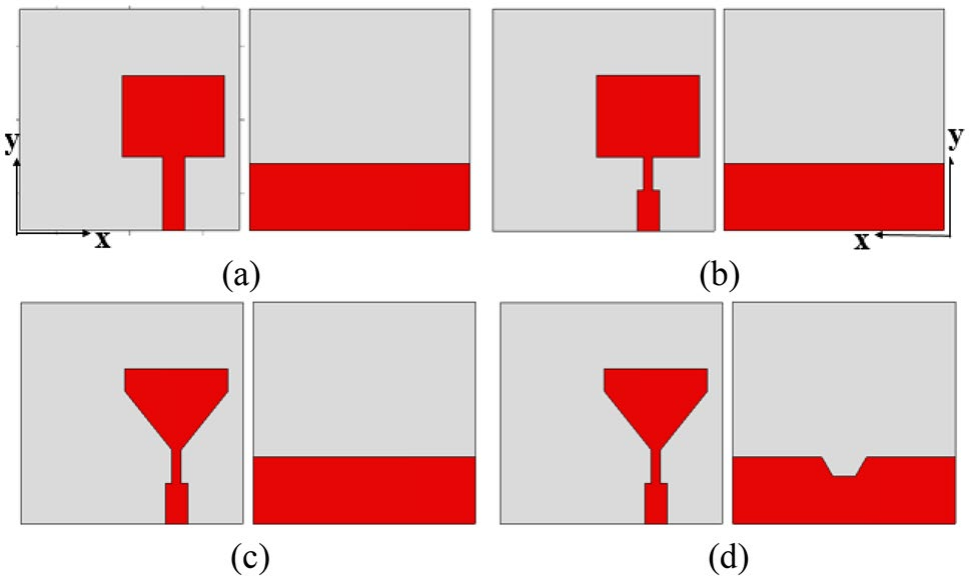
Evolution of the UWB antenna element: (**a**) Antenna-1, (**b**) Antenna-2, (**c**) Antenna-3 and (**d**) Antenna-4.

**Figure 3:**
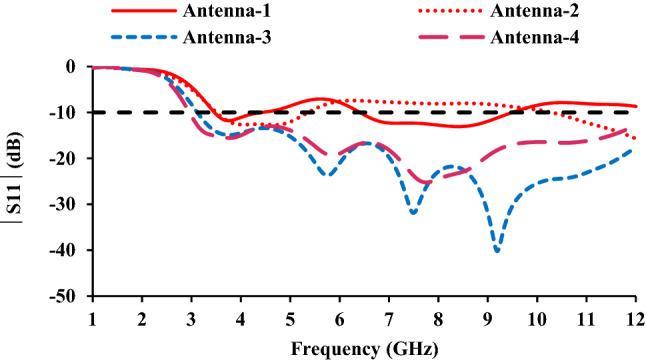
Reflection coefficients of the design steps.

The design parameters of the UWB monopole antenna are given in Table 1. The evolution of the proposed UWB antenna element is depicted in Fig. 2. The length and width of the monopole radiator are optimized to achieve the UWB specifications. The gap between the patch and the ground plane is important for improving radiator performance. The lower corners of the monopole are truncated to improve impedance matching. A hexagonal-shaped defect is introduced in the ground plane to improve impedance matching. The simulated reflection coefficients of the design steps are shown in Fig. 3.

**Table 1:** Antenna parameters.

Parameter	*L*	*q*	*b*	*c*	*h*	*o*
Value (mm)	30	1	16.8	3	11	4
Parameter	*m*	*n*	*k*	*p*	*t*	*u*
Value (mm)	8	5.4	4.5	5.5	20	6

### Integration of bluetooth and GSM bands

**Figure 4:**
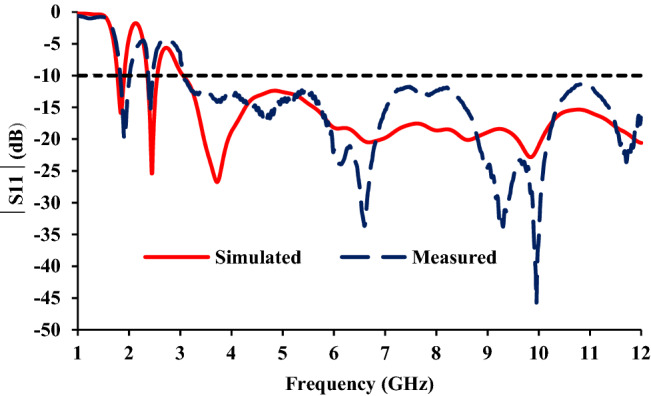
Measured and simulated reflection coefficients of the proposed antenna element.

The ground plane of the antenna element is modified to integrate Bluetooth and GSM bands with the UWB, as shown in Fig. 1b. A stub of length ‘*b*’ is added to the ground plane for Bluetooth (2.4 GHz) resonance. Also, a stub of length (*s* = *t* + *u*) is added to the ground plane for the GSM frequency band. The widths of the stubs are adjusted to improve impedance matching. It is also ensured that the stubs introduced for the additional bands have no significant impact on UWB performance. The measured and simulated reflection coefficients of the antenna element are shown in Fig. 4.

### Equivalent circuit of the proposed antenna

**Figure 5:**
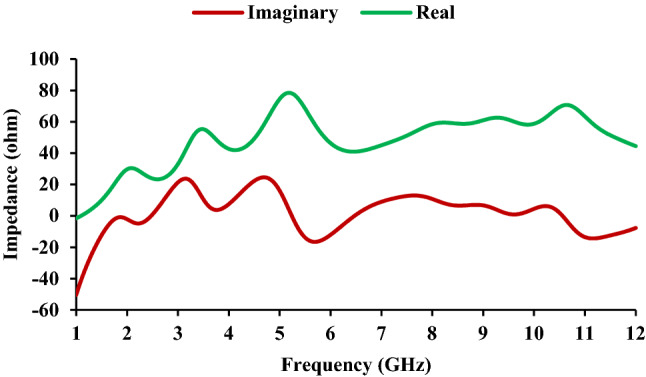
Impedance characteristics of the antenna element.

**Figure 6:**
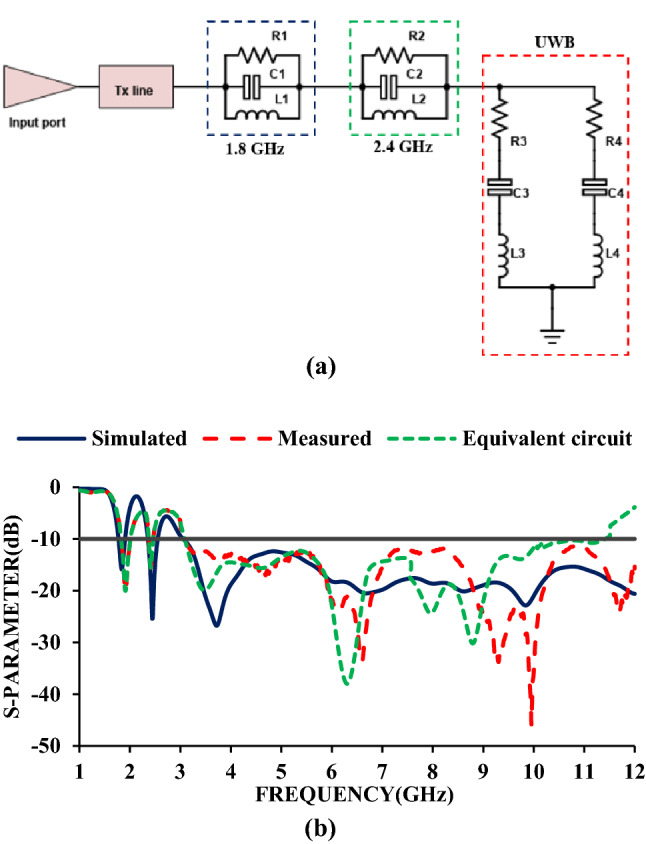
(**a**) Equivalent circuit and (**b**) S-parameters of the equivalent circuit.

The equivalent circuit is used to investigate the physical mechanism of the antenna^25^. The equivalent circuit is calculated using the impedance characteristics, shown in Fig. 5. The two maximum impedance points (3.83 GHz and 9.86 GHz) are selected from the reflection coefficient characteristics, and the corresponding circuit for UWB is derived. When the impedance curve moves from low (negative) to high (positive), a series resonant circuit is drawn, and when the curve moves from high (positive) to low (negative), a parallel resonant circuit is drawn^26^. The equivalent circuit of the antenna is shown in Fig. 6, and the corresponding *RLC* parameters are shown in Table 2. The two parallel resonant circuits correspond to 1.8 GHz and 2.4 GHz, respectively, and the two series resonant circuits correspond to UWB.

**Table 2:** *RLC* parameters of the equivalent circuit.

Frequency (GHz)	*R* (Ω)	*C* (pF)	*L* (nH)
1.8	20.64	0.403	19.38
2.4	28.31	0.112	39.3
3.823	52.91	0.799	2.196
9.865	59.02	1.93	0.137

### Surface current distribution of the antenna

**Figure 7:**
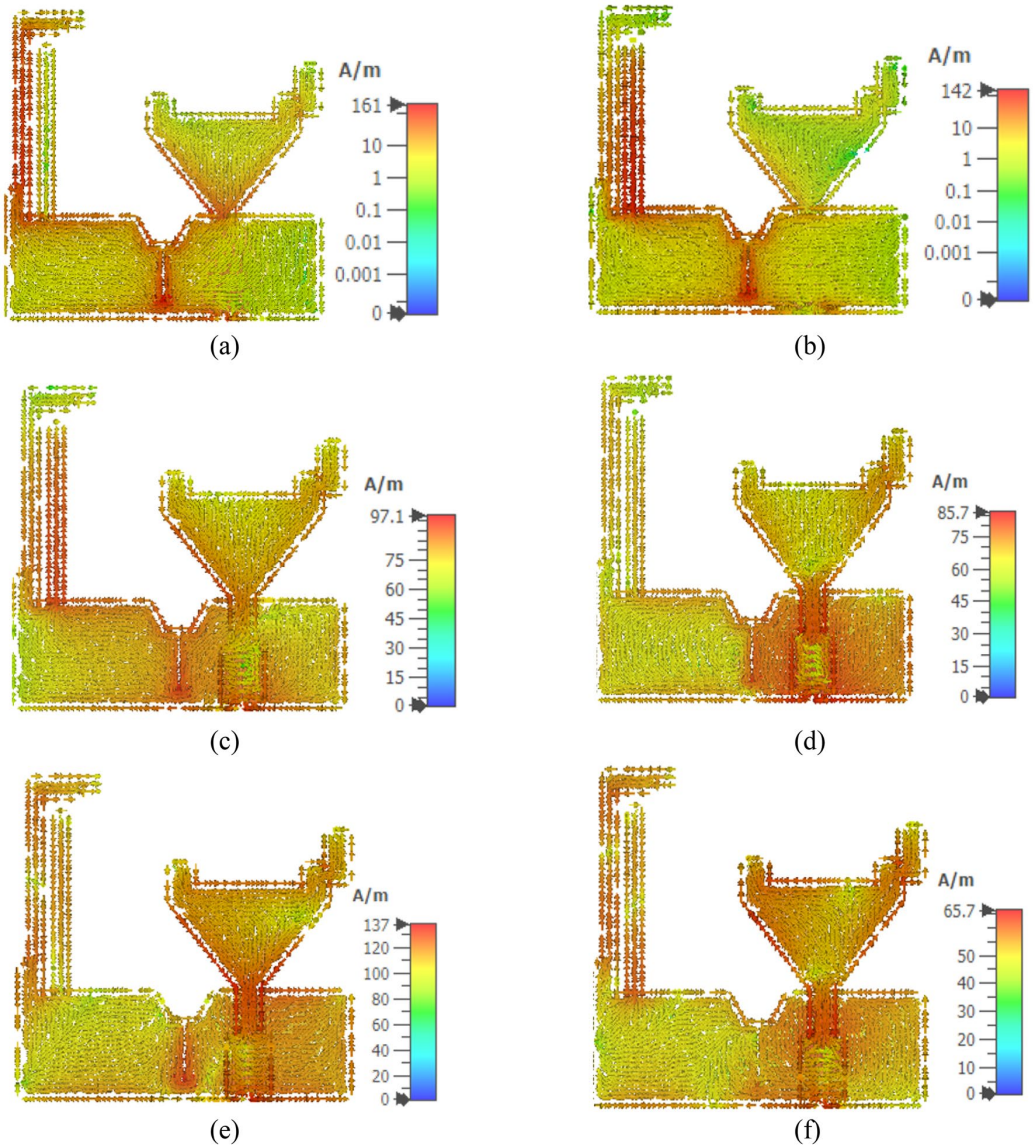
Surface current distribution at (**a**) 1.8 GHz (**b**) 2.4 GHz (**c**) 3.1 GHz (**d**) 5 GHz (**e**) 7 GHz (**f**) 9 GHz.

The surface current is an important parameter to consider as it influences the bandwidth, radiation pattern, and input impedance of the antenna. The surface current distribution of the antenna element at 1.8 GHz, 2.4 GHz, 3.1 GHz, 5 GHz, 7 GHz, and 9 GHz is shown in Fig. 7. Figure 7a, b show the surface current at 1.8 GHz and 2.4 GHz, respectively. The longer stub has a higher current density at 1.8 GHz, while the shorter stub has the highest current density at 2.4 GHz. The surface current distribution for UWB shows that truncation of patch edges aids in higher current density.

## Development of the MIMO antenna

**Figure 8:**
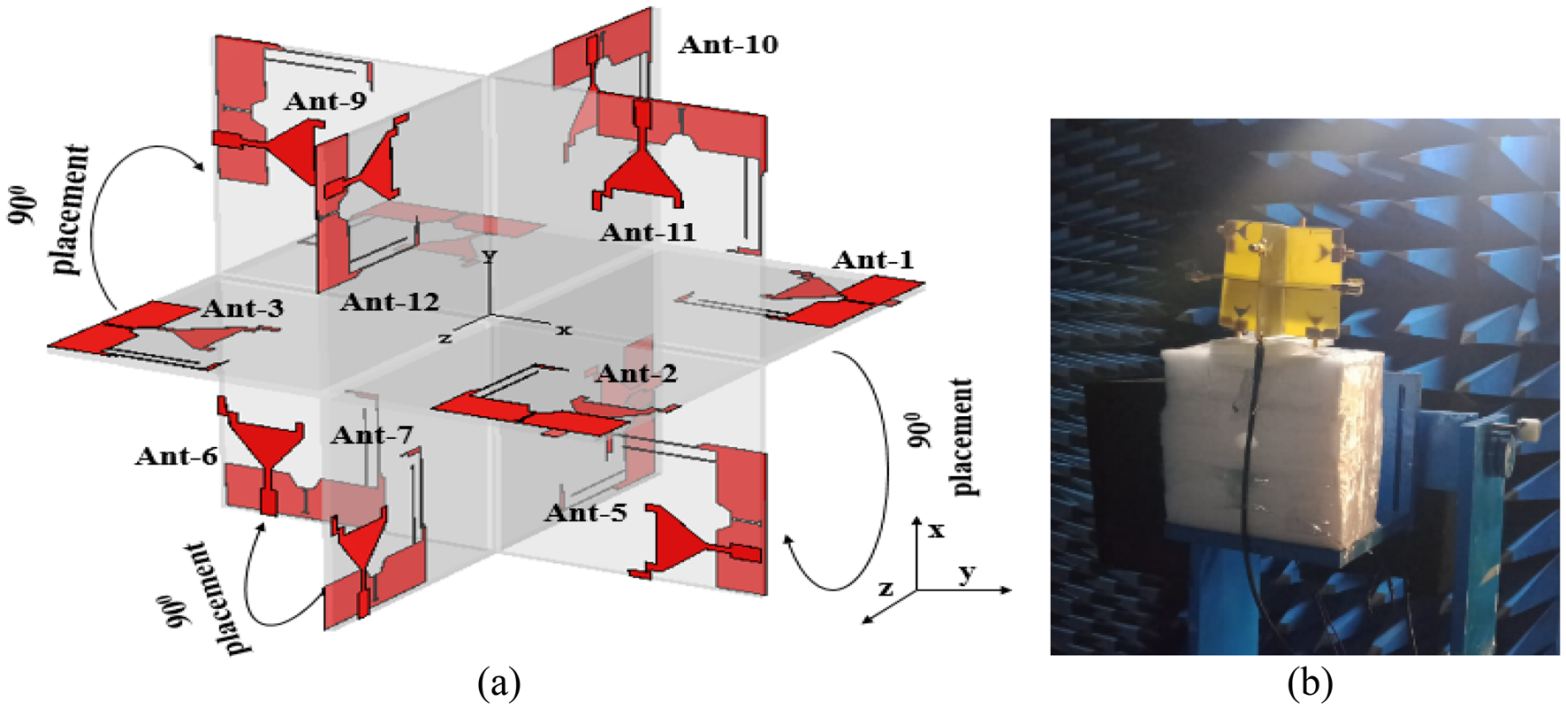
Proposed twelve-port MIMO antenna: (**a**) layout and (**b**) measurement of the fabricated prototype in an anechoic chamber.

The proposed twelve-port MIMO antenna configuration is depicted in Fig. 8a. The antenna is created by arranging four elements in the horizontal plane and eight elements in the vertical plane. The two vertical planes, each with four elements, are arranged in a cross configuration with the horizontal plane. The overall size of the antenna is 100 × 100 mm^2^. Inter-element isolation can be improved by increasing the distance between the antenna elements or by using a decoupling structure between them^27^.

The spacing between the resonating elements is 0.24*λ*_0_ to achieve better isolation. In comparison to the conventional 2-D arrangement, the 3-D orientation of the radiators provides polarization flexibility. When the radiators are oriented in opposite directions, the correlation between them decreases, and the isolation increases. As a result, the MIMO antenna prototype provides polarization diversity while also increasing reliability.

## Fabrication and measurement

The antenna element and MIMO antenna are fabricated in order to test their performance. The Anritsu MS2037C VNA is used to test the *S*-parameters of the twelve-port MIMO antenna.

### S-parameters

**Figure 9:**
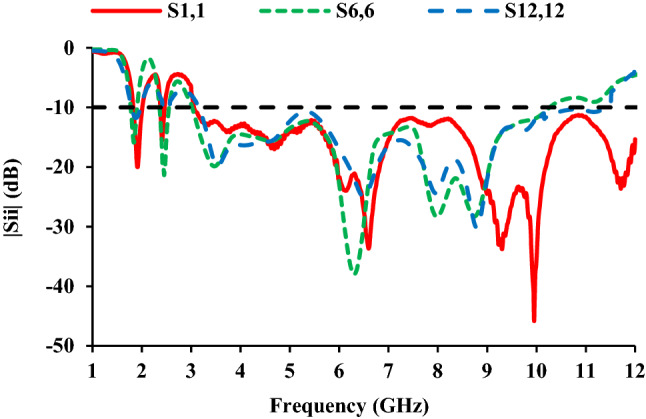
Measured reflection coefficients of the twelve-port MIMO antenna.

**Figure 10:**
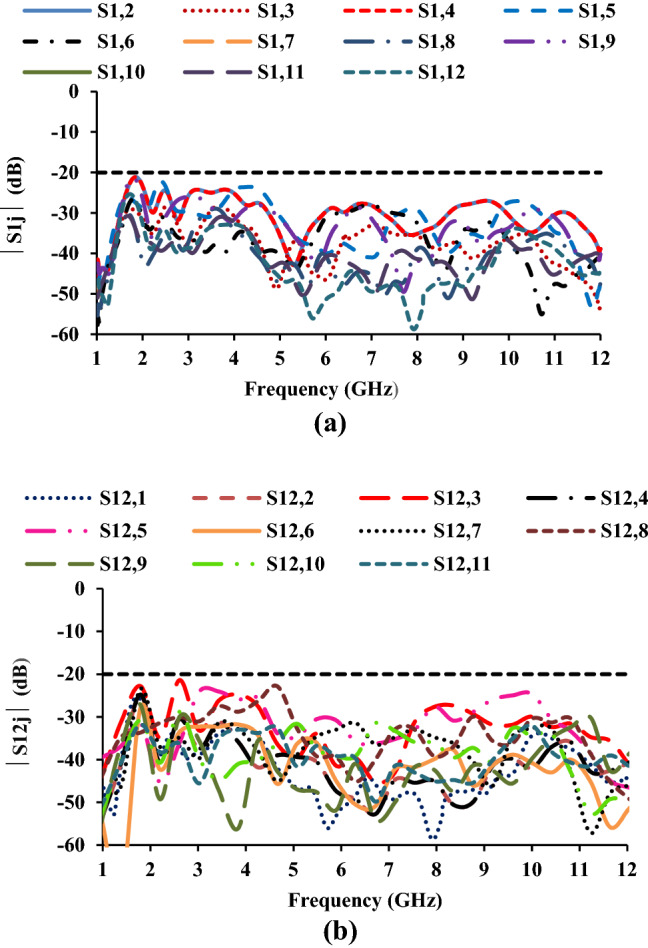
Measured mutual coupling of the twelve-port MIMO antenna: (**a**) with respect to port-1 and (**b**) with respect to port-12.

The measured *S*-parameters of the twelve-port MIMO antenna are shown in Figs. 9 and 10. The *S*-parameters (*S*_11_, *S*_66_, and *S*_1212_) are measured at port-1 in the horizontal plane, and port-6 and port-12 in the vertical planes. The *S*_*ii*_ characteristics show that the antenna has a good impedance over the UWB, GSM, and Bluetooth frequencies.

The mutual coupling characteristics of the proposed twelve-port MIMO antenna are depicted in Fig. 10. The *S*_*ij*_ characteristics illustrate that the antenna elements offer isolation greater than 20 dB.

### Radiation performance

**Figure 11:**
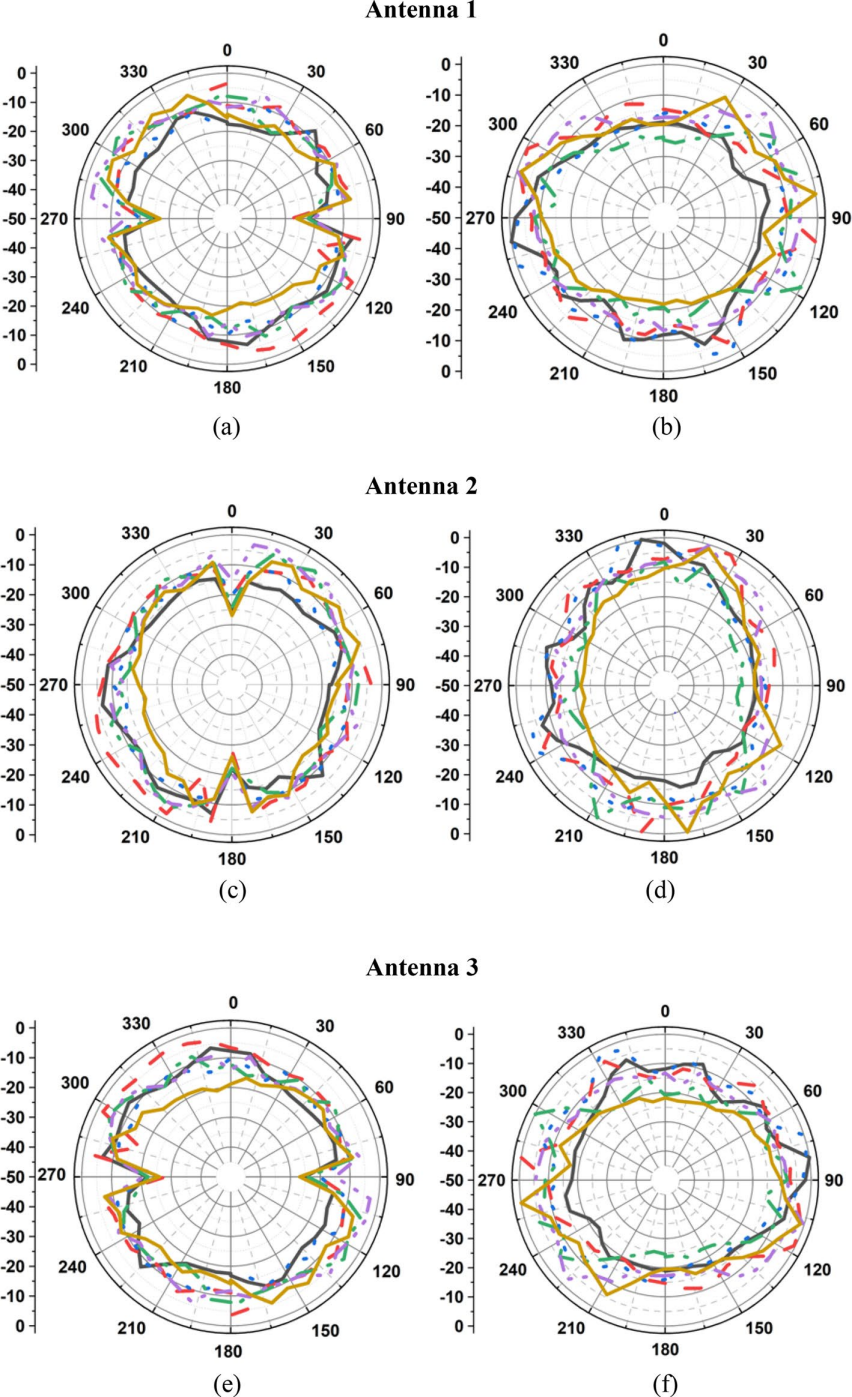

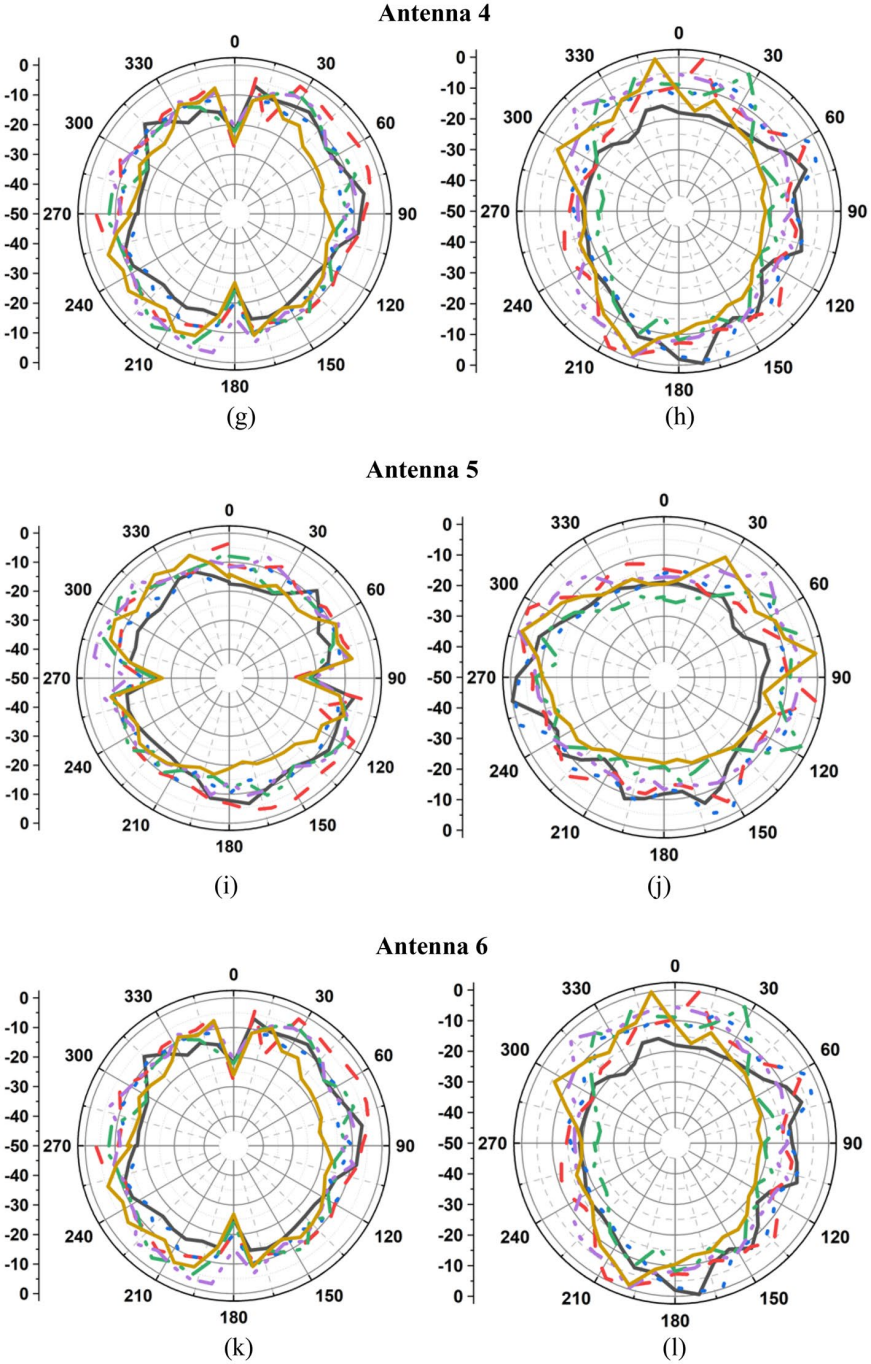

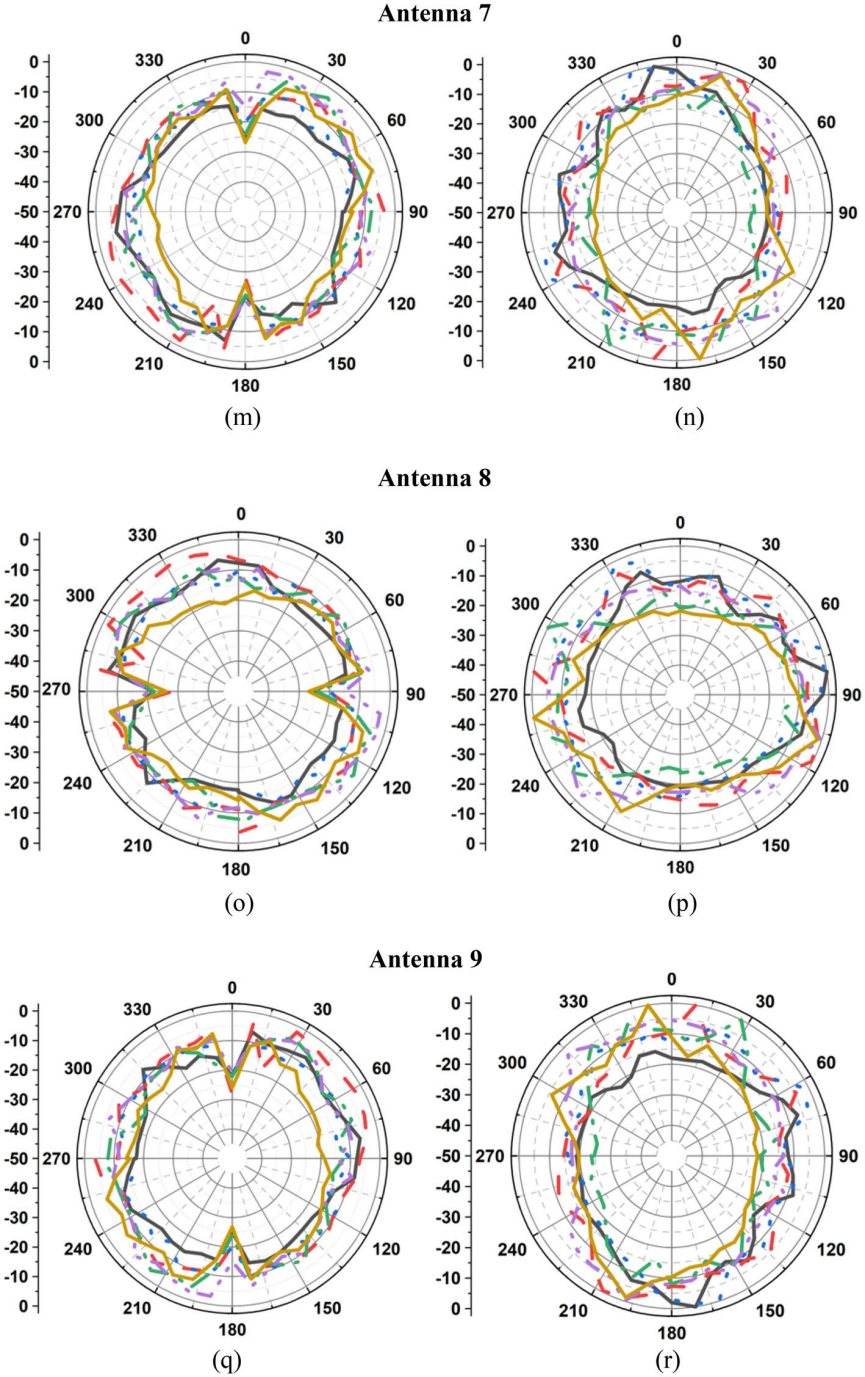

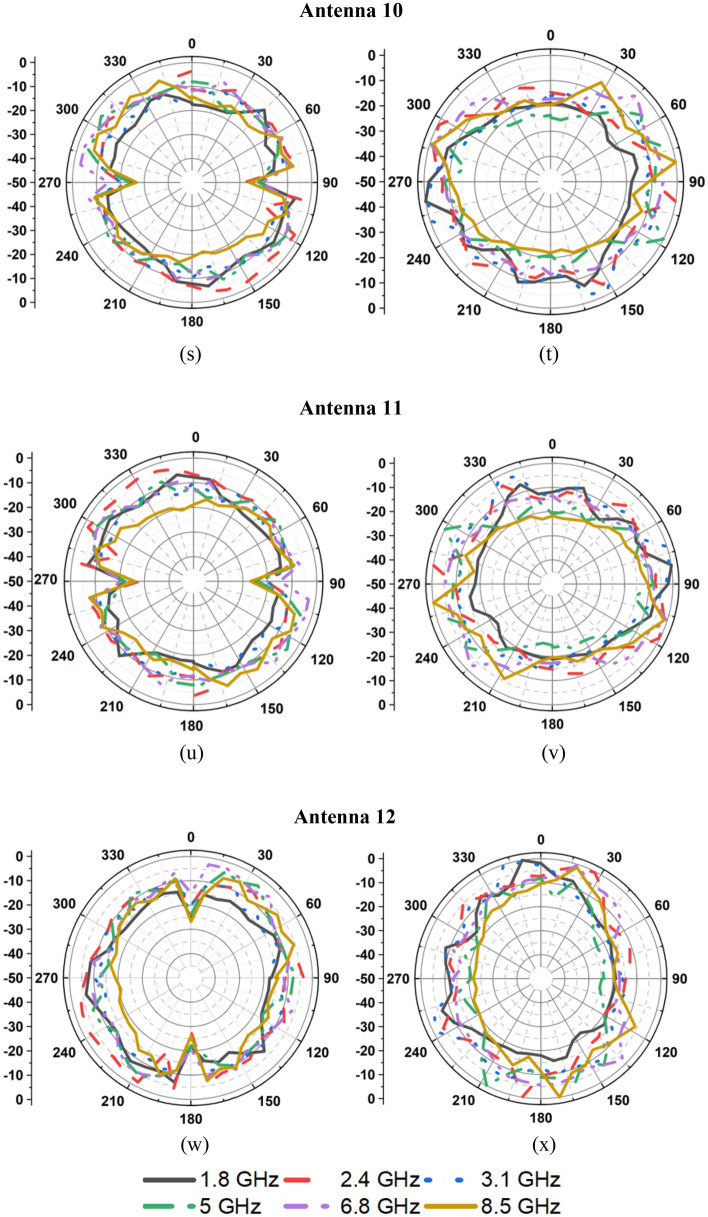
Measured radiations pattern at 1.8 GHz, 2.4 GHz, 3.1 GHz, 5 GHz, 6.8 GHz, 8.5 GHz: (**a**) E-plane/*yz*/*φ* = 90°, (**b**) H-plane/*xz*/*φ* = 0°, (**c**) E-plane/*yz*/*φ* = 90°, (**d**) H-plane/*xz*/*φ* = 0°, (**e**) E-plane/*yz*/*φ* = 90°, (**f**) H-plane/*xz*/*φ* = 0°, (**g**) E-plane/*yz*/*φ* = 90°, (**h**) H-plane/*xz*/*φ* = 0°, (**i**) E-plane/*yz*/*φ* = 90°, (**j**) H-plane/*xz*/*φ* = 0°, (**k**) E-plane/*yz*/*φ* = 90°, (**l**) H-plane/*xz*/*φ* = 0°, (**m**) E-plane/*yz*/*φ* = 90°, (**n**) H-plane/*xy*/*φ* = 0°, (**o**) E-plane/*xz*/*φ* = 90°, (**p**) H-plane/*xy*/*φ* = 0°, (**q**) E-plane/*yz*/*φ* = 90°, (**r**) H-plane/*xy*/*φ* = 0°, (**s**) E-plane/*xz*/*φ* = 90°, (**t**) H-plane/*xy*/*φ* = 0°, (**u**) E-plane/*yz*/*φ* = 90°, (**v**) H-plane/*xy*/*φ* = 0°, (**w**) E-plane/*xz*/*φ* = 90°, (**x**) H-plane/*xy*/*φ* = 0°.

**Figure 12:**
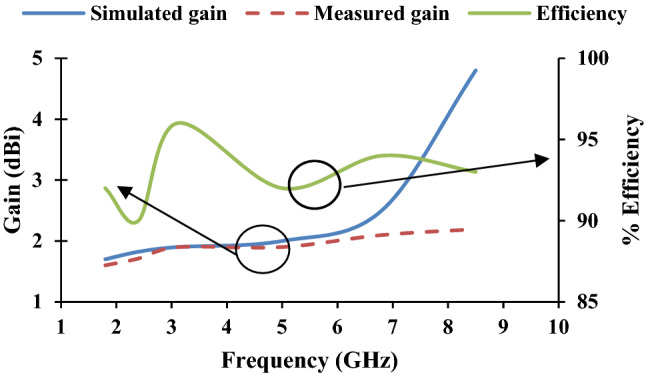
Gain and efficiency of the proposed antenna.

The measured radiation patterns of the twelve-port MIMO antenna at 1.8 GHz, 2.4 GHz, 3.1 GHz, 5 GHz, 6.8 GHz, and 8.5 GHz are depicted in Fig. 11. The radiation performance of the fabricated prototype is measured in an anechoic chamber as depicted in Fig. 8b. Figure 12 presents the measured gain and efficiency of the prototype antenna. The gain and efficiency of the proposed antenna are greater than 1.6 dBi and 90%, respectively.

### Diversity performance

**Figure 13:**
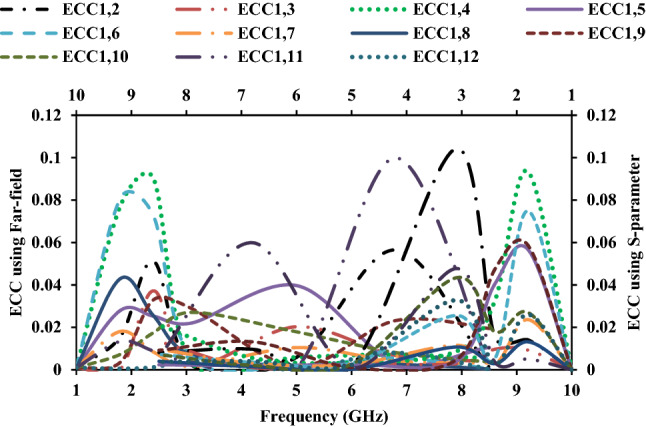
ECC of the MIMO antenna with respect to port-1.

**Figure 14:**
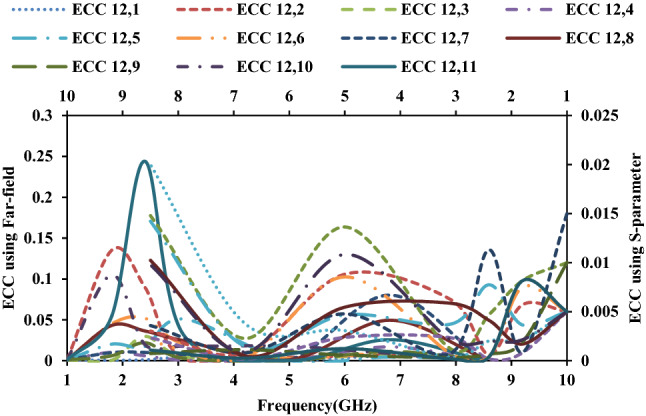
ECC of the MIMO antenna with respect to port-12.

The diversity performance of the twelve-port MIMO antenna is estimated using metrics such as envelope correlation coefficient (ECC), diversity gain (DG), mean effective gain (MEG), total active reflection coefficient (TARC), and channel capacity loss (CCL). The ECC value should ideally be zero, but in practice it is < 0.5. ECC can be calculated using the *S*-parameter or the far-field, respectively, using Eqs. (4) and (5).

4 $$ECC\left( {\rho_{e} } \right) = \frac{{\left| {S_{ii}^{*} S_{ij} + S_{ji}^{*} S_{jj} } \right|^{2} }}{{\left( {1 - \left| {S_{ii} } \right|^{2} - \left| {S_{ij} } \right|^{2} } \right)\left( {1 - \left| {S_{ji} } \right|^{2} - \left| {S_{ii} } \right|^{2} } \right)}}$$

5 $$ECC\left( {\rho_{e} } \right) = \frac{{\left| {\iint {\left[ {\overrightarrow {{F_{1} }} \left( {\theta ,\varphi } \right).\overrightarrow {{F_{2} }} \left( {\theta ,\varphi } \right)} \right]d\Omega }} \right|^{2} }}{{\iint {\left| {\overrightarrow {{F_{1 } }} \left( {\theta ,\varphi } \right)} \right|^{2} }d\Omega \iint {\left| {\overrightarrow {{F_{2} }} \left( {\theta ,\varphi } \right)} \right|^{2} }d\Omega }}$$

where *S*_*ij*_ denotes the *S*-parameter of antenna *i* in relation to antenna *j*, *F*_*i*_ is the field radiated by the antenna. The calculated ECC values show that the antenna elements are less correlated, as shown in Figs. 13 and 14.

The two types of diversity gain are apparent diversity gain (ADG) and effective diversity gain (EDG), which are calculated using the Eqs. (6) and (7), respectively. They differ in the way that EDG includes efficiency while ADG does not. The practical limit for DG is > 9.9 dB. The ADG and EDG are calculated using the far-field and *S*-parameters, and they meet the practical limit for DG. Tables 3 and 4 present the ADG and EDG of the proposed MIMO antenna in relation to port-1 and port-12, respectively.

6 $$ADG{ } = 10\sqrt {1 - \left| {\rho_{e} } \right|^{2} }$$

7 $$EDG{ } = \eta_{total} *{\text{ADG}}$$

where

$$\eta_{total} = \eta_{irad} \left( {1 - \mathop \sum \limits_{j = 1}^{M} \left| {S_{ij} } \right|^{2 } } \right)$$

$$\eta_{irad} = \left( {1 - \mathop \sum \limits_{j = 1}^{M} \left| {S_{ij} } \right|} \right)$$

**Table 3:** ADG and EDG of the proposed antenna in relation to port-1.

Parameter	Frequency (GHz)	Port										
		1 and 2	1 and 3	1 and 4	1 and 5	1 and 6	1 and 7	1 and 8	1 and 9	1 and 10	1 and 11	1 and 12
ADG (far-field)	1.8	9.997	9.997	9.970	9.996	9.967	9.998	9.990	9.997	9.998	9.998	9.998
	2.4	9.986	9.993	9.959	9.991	9.973	9.998	9.996	9.994	9.998	9.999	9.999
	3.1	9.945	9.999	9.999	9.997	9.996	9.999	9.999	9.995	9.996	9.988	9.994
	5	9.998	9.997	9.999	9.992	9.999	9.999	9.999	9.997	9.995	9.999	9.999
	6.8	9.984	9.998	9.999	9.999	9.999	9.999	9.998	9.997	9.999	9.949	9.999
	8.5	9.999	9.999	9.997	9.984	9.989	9.999	9.999	9.998	9.998	9.982	9.999
ADG (*S*-parameter)	1.8	9.998	9.998	9.99	9.998	9.998	9.998	9.998	9.999	9.999	9.999	9.999
	2.4	9.998	9.999	9.994	9.996	9.999	9.999	9.999	9.988	9.998	10	10
	3.1	9.999	10	10	9.999	9.999	10	9.999	9.999	9.999	9.999	9.999
	5	9.999	9.999	9.999	9.999	9.999	9.999	9.999	9.999	9.998	9.999	9.999
	6.8	9.999	9.999	10	10	9.999	10	9.999	9.999	10	9.999	10
	8.5	10	10	9.999	9.999	9.999	10	9.999	9.999	9.999	9.99	10
EDG (far-field)	1.8	9.059	9.058	9.033	9.057	9.031	9.053	9.052	9.060	9.050	9.051	9.050
	2.4	9.492	9.498	9.466	9.481	9.479	9.504	9.501	9.499	9.503	9.504	9.504
	3.1	9.749	9.790	9.790	9.790	9.799	9.790	9.789	9.795	9.790	9.788	9.794
	5	9.136	9.135	9.136	9.129	9.136	9.136	9.136	9.136	9.135	9.136	9.136
	6.8	9.896	9.911	9.911	9.911	9.911	9.911	9.911	9.909	9.911	9.862	9.911
	8.5	9.166	9.165	9.164	9.165	9.166	9.165	9.166	9.164	9.161	9.164	9.166
EDG (*S*-parameter)	1.8	9.1	9.1	9.061	9.060	9.060	9.059	9.053	9.061	9.053	9.053	9.06
	2.4	9.503	9.52	9.51	9.49	9.504	9.52	9.504	9.49	9.503	9.52	9.52
	3.1	9.796	9.799	9.799	9.797	9.796	9.799	9.799	9.799	9.796	9.799	9.799
	5	9.138	9.136	9.137	9.137	9.137	9.137	9.137	9.137	9.136	9.136	9.146
	6.8	9.999	9.98	9.98	9.98	9.98	9.99	9.99	9.995	9.99	9.97	9.999
	8.5	9.166	9.166	9.166	9.166	9.166	9.166	9.167	9.168	9.166	9.166	9.166

**Table 4:** ADG and EDG of the proposed antenna in relation to port-12.

Parameter	Frequency (GHz)	Port										
		12 and 1	12 and 2	12 and 3	12 and 4	12 and 5	12 and 6	12 and 7	12 and 8	12 and 9	12 and 10	12 and 11
ADG (far-field)	1.8	9.9	9.999	9.999	9.969	9.997	9.899	9.989	9.999	9.999	9.999	9.998
	2.4	9.999	9.908	9.999	9.989	9.996	9.989	9.999	9.990	9.999	9.944	9.985
	3.1	9.999	9.959	9.995	9.999	9.998	9.986	9.999	9.993	9.997	9.998	9.697
	5	9.999	9.999	9.989	9.999	9.986	9.996	9.999	9.996	9.999	9.994	9.995
	6.8	9.989	9.999	9.999	9.999	9.991	9.998	9.998	9.989	9.999	9.998	9.999
	8.5	9.997	9.999	9.987	9.998	9.998	9.999	9.967	9.987	9.997	9.999	9.996
ADG (*S*-parameter)	1.8	9.999	10	10	9.999	9.999	9.999	9.999	10	10	10	9.999
	2.4	10	9.999	10	9.999	9.997	9.999	10	9.999	10	9.999	9.999
	3.1	10	9.999	9.999	10	9.999	9.999	10	9.999	9.999	9.999	9.999
	5	10	10	9.999	10	9.999	9.997	10	9.999	10	9.998	9.999
	6.8	9.999	10	10	10	9.998	9.999	9.999	9.999	10	9.999	10
	8.5	9.998	10	9.998	9.999	9.999	10	9.999	9.998	9.999	10	9.999
EDG (far-field)	1.8	9.459	9.372	9.459	9.458	9.457	9.449	9.459	9.450	9.459	9.407	9.445
	2.4	9.416	9.379	9.413	9.417	9.416	9.405	9.417	9.411	9.415	9.416	9.133
	3.1	8.964	8.964	8.964	8.964	8.953	8.962	8.964	8.962	8.964	8.963	8.960
	5	9.132	9.133	9.104	9.124	9.044	9.124	9.114	9.124	9.133	9.133	9.134
	6.8	9.972	9.971	9.972	9.971	9.972	9.972	9.940	9.960	9.971	9.970	9.969
	8.5	9.850	9.851	9.851	9.852	9.852	9.852	9.852	9.852	9.852	9.852	9.852
EDG (*S*-parameter)	1.8	9.459	9.45	9.459	9.459	9.46	9.459	9.46	9.452	9.459	9.459	9.459
	2.4	9.417	9.418	9.418	9.418	9.41	9.418	9.417	9.417	9.417	9.417	9.417
	3.1	8.964	8.964	8.965	8.965	8.965	8.965	8.964	8.964	8.965	8.964	8.964
	5	9.134	9.134	9.133	9.134	9.13	9.134	9.134	9.134	9.134	9.134	9.135
	6.8	9.973	9.972	9.973	9.973	9.972	9.973	9.973	9.972	9.972	9.972	9.973
	8.5	9.853	9.852	9.852	9.853	9.852	9.853	9.855	9.854	9.853	9.854	9.853

MEG quantifies the ability of the antenna to receive transmitted electromagnetic power. MEG can be calculated using the far-field Eq. (8).

8 $$MEG = \mathop \smallint \limits_{0}^{2\pi } \mathop \smallint \limits_{0}^{\pi } \left[ {\frac{XPR}{{1 + XPR}}G_{\theta } \left( {\theta ,\emptyset } \right)P_{\theta } \left( {\theta ,\emptyset } \right) + \frac{1}{1 + XPR}G_{\emptyset } \left( {\theta ,\emptyset } \right)P_{\emptyset } \left( {\theta ,\emptyset } \right)} \right]\sin \theta d\theta d\emptyset$$

Ideally, the MEG difference should be less than 3 dB. The proposed MIMO antenna has a MEG difference of less than 1 dB.

**Figure 15:**
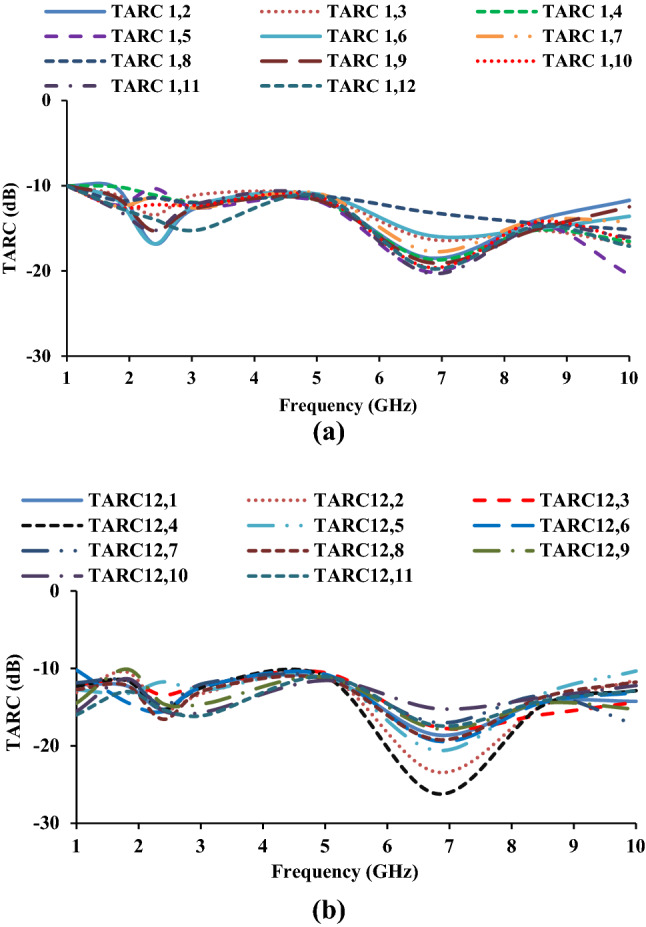
TARC of the MIMO antenna: (**a**) with respect to port-1, (**b**) with respect to port-12.

TARC is another metric used to determine the impact of one antenna element on another. TARC is defined as the square root of the total reflected power divided by the total incident power, as shown in Eq. (9).

9 $$TARC = \frac{{\sqrt {\mathop \sum \nolimits_{i = 1}^{N} \left| {b_{i} } \right|^{2} } }}{{\sqrt {\mathop \sum \nolimits_{i = 1}^{N} \left| {a_{i} } \right|^{2} } }}$$

where *a*_*i*_ is the incident signal and *b*_*i*_ is the received signal. Figure 15 depicts the TARC of the MIMO antenna in relation to port-1 and port-12. The calculated results show that the lower the TARC value, the lower the mutual coupling.

CCL is used to investigate capacity loss due to correlation in MIMO channels. The CCL of a MIMO system can be calculated as

10 $$CCL = - \log_{2} \left| {\Psi^{R} } \right|$$

**Figure 16:**
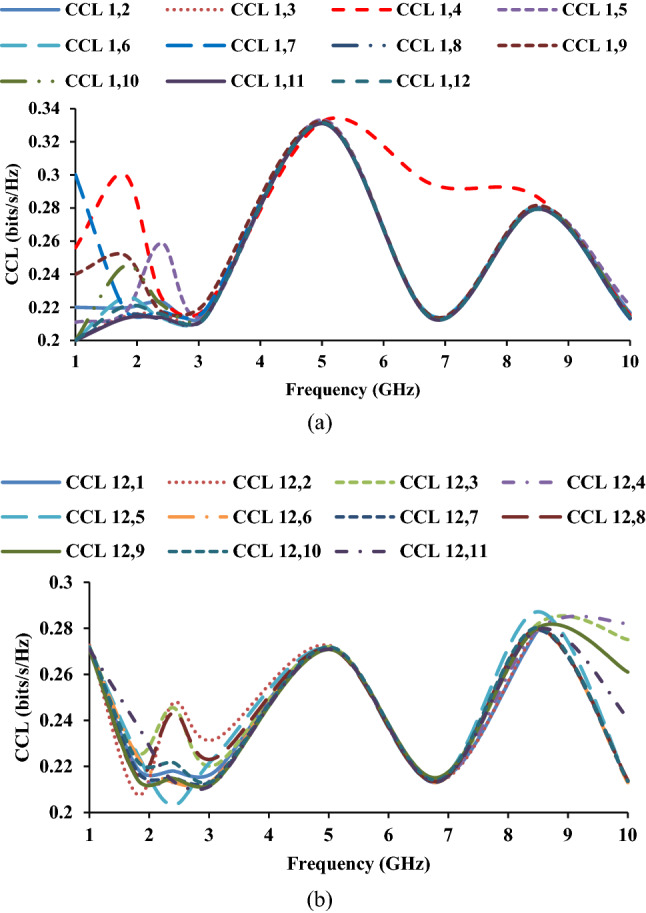
CCL of the MIMO antenna: (**a**) with respect to port-1 and (**b**) with respect to port-12.

Figure 16 depicts the CCL of the MIMO antenna in relation to port-1 and port-12.

The correlation matrix of the receiving antenna is given by

11 $$\Psi^{R} = \left[ {\begin{array}{*{20}c} {\rho_{11} } & {\rho_{12} } \\ {\rho_{21} } & {\rho_{22} } \\ \end{array} } \right]$$

where $$\rho_{11} = \left( {1 - \left| {S_{11} } \right|^{2} - \left| {S_{12} } \right|^{2} } \right),$$ $$\rho_{22} = \left( {1 - \left| {S_{22} } \right|^{2} - \left| {S_{21} } \right|^{2} } \right)$$.

$$\rho_{12} = - \left( {S_{11}^{*} S_{12} + S_{21}^{*} S_{12} } \right),$$ and $$\rho_{21} = - \left( {S_{22}^{*} S_{21} + S_{12}^{*} S_{21} } \right)$$.

The practical limit of CCL is 0.4 bits/s/Hz, and the proposed antenna offers CCL less than 0.35 bits/s/Hz.

**Figure 17:**
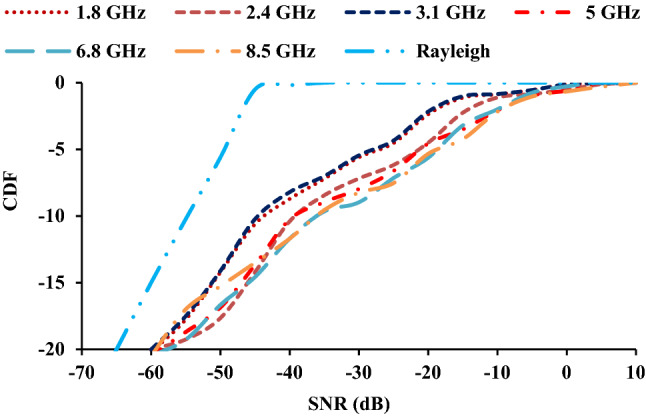
CDF of the twelve-port MIMO antenna.

Maximal ratio combining (MRC) and selection combining (SC) are diversity combining techniques that combine the signals received from the antenna to increase the mean signal to noise ratio (SNR) and yield reliability in fading environments. The Eq. (12) can be used to calculate the cumulative distribution function (CDF) of the MIMO antenna under the rayleigh condition^28^. Figure 17 shows that the twelve-port configuration outperforms the two-element case in terms of diversity performance.

12 $$F_{MRC} \left( \gamma \right) = 1 - \mathop \sum \limits_{i = 1}^{K} \left( {\frac{{\lambda_{i}^{K - 1} e^{{\left( {\frac{ - x}{{\lambda_{i} }}} \right)}} }}{{\mathop \prod \nolimits_{j \ne i}^{K} \left( {\lambda_{i} - \lambda_{j} } \right)}}} \right)$$

where *λ* is the eigen value obtained from the signal covariance matrix (*Λ*_*MRC*_) and *K* is the number of antenna elements. The covariance matrix is given by Eq. (13).

13 $$\Lambda_{MRC} = \rho_{e} \sqrt {MEG_{i} MEG_{j} }$$

The CDF of the Rayleigh is calculated using Eq. (14), where *Г* is the average SNR.

14 $$F_{Rayleigh} (\gamma ) = 1 - e^{{ - \left( {\frac{x}{\Gamma}} \right)}}$$

## Antenna housing effects

The location of the antenna in the vehicle has a significant impact on its performance. The proposed antenna can be mounted on the roof of a car using a shark fin mount or integrated into the existing printed circuit board. The proposed automotive antenna can be installed on the roof of a car through the chassis cavity^29^. For automotive communications, the antenna housing effect is discussed in order to evaluate antenna performance in the presence of metallic conductors^30–32^.

A metal plate is used to mimic the car roof to investigate the effects of antenna housing. The size of the metal plate ranges from 40 × 40 × 5 cm^3^ to 80 × 80 × 5 cm^3^.

**Figure 18:**
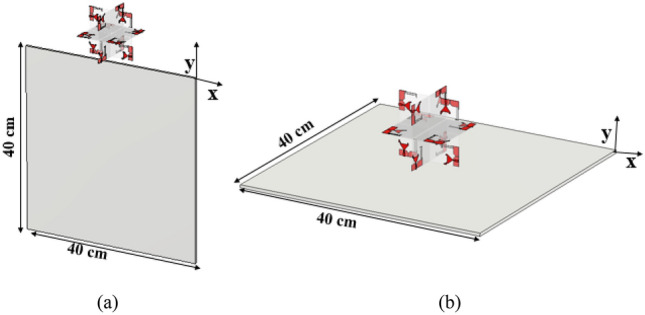
Housing effect: (**a**) case-1 and (**b**) case-2.

**Figure 19:**
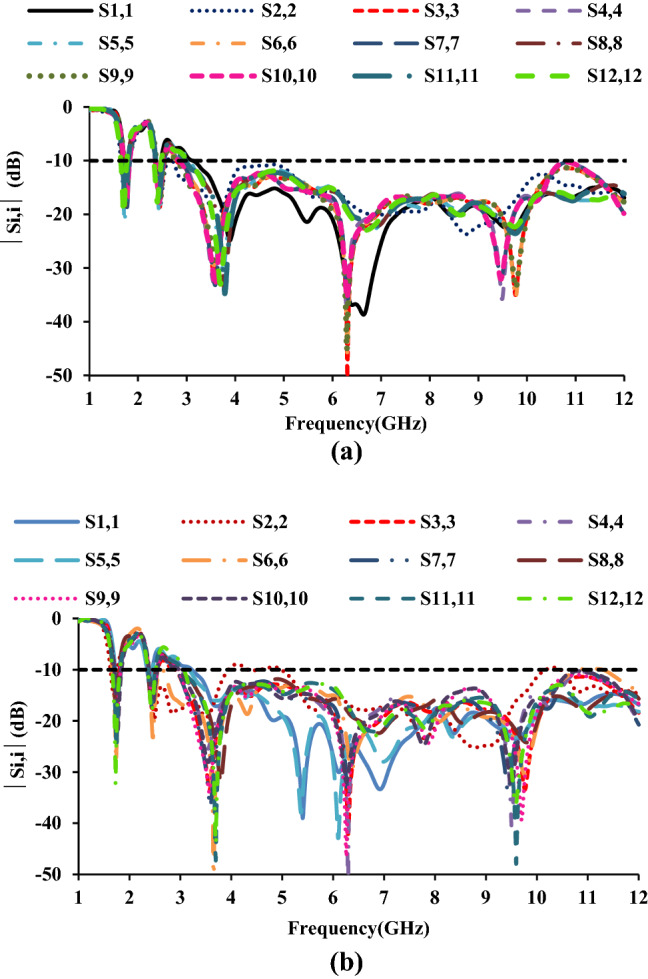
Effect of housing on the performance of the proposed antenna: (**a**) case-1 and (**b**) case-2.

Two scenarios are considered when studying the effects of antenna housing. The antenna is positioned in the *xz*- and *yz*-planes as shown in Fig. 18. In the *xz*-plane, the antenna is perpendicular to the metal conductor, while in the *yz*-plane, the antenna is to the side of the metal conductor. The omnidirectional characteristic is influenced if the antenna is placed at the top of the *yz*-plane. Figure 19 depicts the simulated reflection coefficients of the twelve-port antenna when antenna housing effects are taken into account. The simulation results show that the presence of a metal conductor has no significant effect on the antenna characteristic in either scenario. The presence of a metal plate has no effect on the *xz*-plane. Even in the presence of a metal plate, the antenna maintains its omnidirectional behavior.

**Figure 20:**
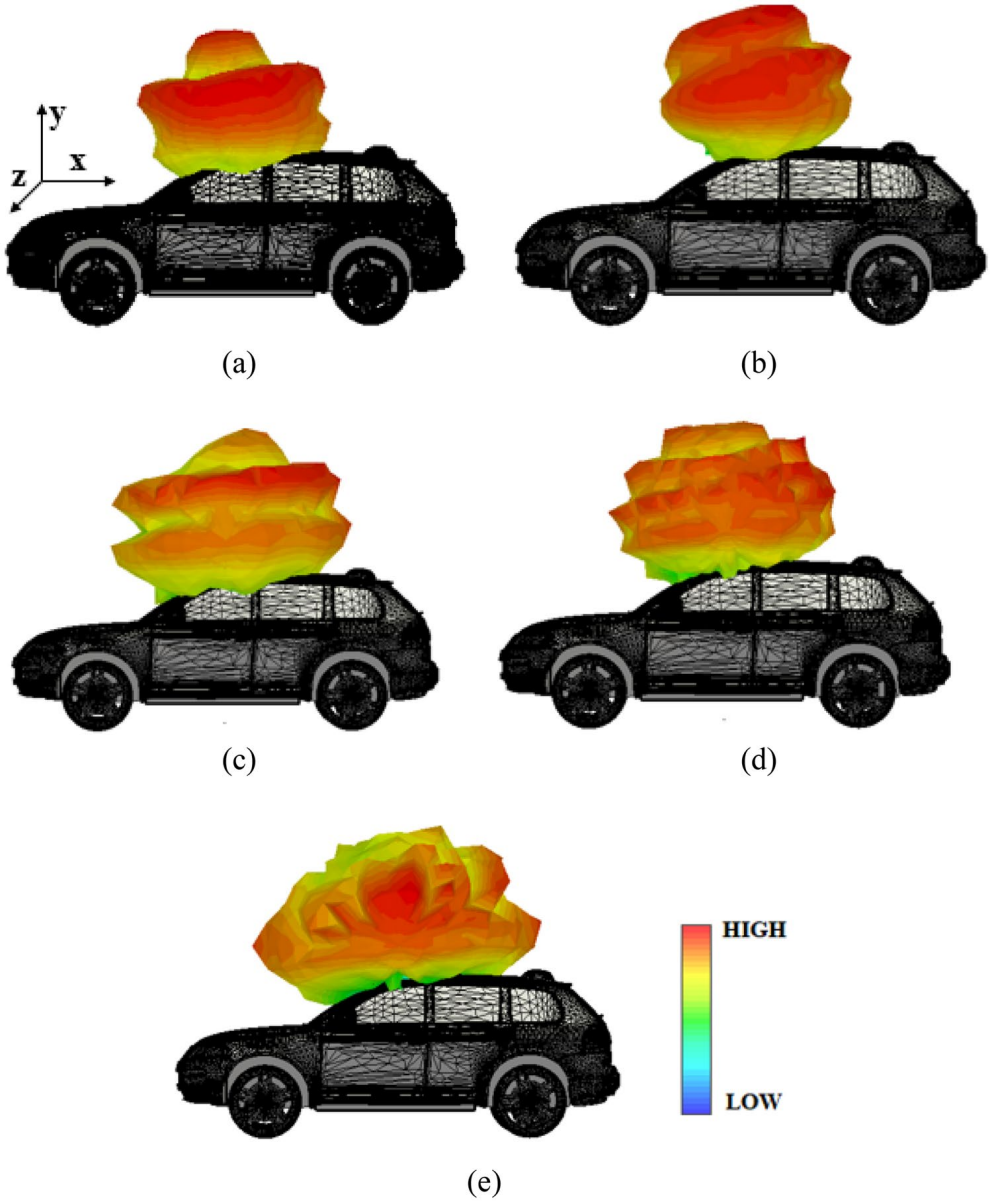
On-car performance of the proposed antenna: (**a**) 1.8 GHz, (**b**) 2.4 GHz, (**c**) 3.1 GHz, (**d**) 6.8 GHz, (**e**) 8.5 GHz.

The asymptotic solver in CST is used to estimate the far-field performance of the proposed antenna when integrated with a vehicle. An open-source CAD model of the Volkswagen Touareg is used for estimating the far-field characteristics. The on-car performance of the proposed antenna is depicted in Fig. 20. The results imply that the antenna exhibits omnidirectional characteristics when placed on the body of the vehicle. The directivity is greater than 6 dB for all observed frequencies.

Table 5 compares the reported and proposed MIMO antenna designs. The main advantages of the proposed antenna are:

1. In comparison to the antenna structures^7,14,20,33–59^, the proposed antenna geometry has twelve-elements, and covers two narrow bands (GSM and Bluetooth) and the entire UWB.
2. The proposed antenna achieves diversity by using 3-D orientations, whereas 2-D orientations were used in^7,14,20,33–48,50–53,57–59^.
3. Unlike the antenna structures reported in^14,20,34,36–48,50–59^, the proposed MIMO antenna configuration occupies less area while having a larger number of resonating elements. The antennas in^7,33,35,47^ occupied an equivalent/smaller area but had fewer elements.
4. The proposed MIMO antenna outperforms in terms of ECC, DG, MEG, TARC, and CCL, whereas all of these diversity factors were not investigated in the majority of reported papers^14,20,33–38,40,41,43–45,47,49,51–55,58,59^.
5. The housing effect and on-car body performance of the proposed 3-D MIMO antenna are investigated, whereas they were previously studied only for single-element/two-element/2-D MIMO antenna designs^1,31,32,45,55^.

**Table 5:** Comparison of the proposed work to previous literature.

Refs.	Size in single plane (*λ*_0_ × *λ*_0_)	Substrate/Thickness (mm)	Number of elements	Bandwidth (GHz)	CCL (bits/s/Hz)	Polarization
^7^	0.19 × 0.31	FR-4/0.8	2	3.1–10.6	< 0.4	Single
^14^	2.1 × 2.2	FR-4/1.6	4	0.76–1.02, 3.01–12.5	–	Dual
^33^	0.25 × 0.322	FR-4/1.5748	2	2.1–1.4	–	Single
^34^	0.4 × 0.2	FR-4/1.6	2	3–11	–	Single
^35^	0.25 × 0.366	FR-4/1.6	2	2.5–12	–	Single
^36^	0.4 × 0.233	FR-4/1.6	2	2–10	–	Single
^37^	0.93 × 0.93	FR-4/1.6	4	2.4–2.5, 5.1–5.9	–	Single
^38^	0.47 × 0.47	FR-4/1.6	4	3.1–11	–	Circular
^39^	0.6 × 0.6	FR-4/1.6	4	3.0–16.2	< 0.4	Dual
^40^	0.55 × 0.55	FR-4/1.6	4	2.73–10.68	–	Dual
^41^	0.68 × 0.68	FR-4/1.6	4	3.4–3.8	–	Circular
^42^	0.29 × 0.29	FR-4/1.6	4	2.3–13.75	< 0.2	Dual
^43^	0.383 × 0.383	Taconic/0.8	4	3–13.2	–	Dual
^44^	0.283 × 0.283	FR-4/1.6	4	2.5–12	–	Dual
^45^	0.325 × 0.325	FR-4/1	4	1.95–6.25	–	Single
^46^	0.56 × 0.56	FR-4/1.6	4	2.1–20	< 0.4	Dual
^47^	0.268 × 0.138	FR-4/0.8	4	2.3–12	–	Single
^48^	0.644 × 0.518	FR-4/1.6	4	2.4–2.52, 3.66–4, 4.62–5.54	< 0.4	Dual
^49^	0.33 × 0.37 (2D)	FR-4/0.8	4	3.1–10.6	–	Dual
	0.217 × 0.217 (3D)	FR-4/0.8	4	3.1–10.6	–	Single
^50^	0.62 × 0.62	FR-4/1	8	3.1–10.6	< 0.5	Dual
^51^	0.88 × 0.88	FR-4/0.8	8	3.1–10.6	–	Dual
^52^	0.58 × 1.16	FR-4/1.6	8	2.55–2.65	–	Dual
^53^	1.7 × 0.85	FR-4/0.8	8	3.4–3.6, 4.8–5.1	–	Single
^54^	0.68 × 0.68	FR-4/1.6	8	2.9–12	–	Triple
^55^	0.72 × 0.72	FR-4/1.6	8	2.4–12	–	Triple
^56^	0.33 × 0.33	FR-4/1.6	8	2–12	< 0.3	Quad
^57^	0.83 × 1.7	FR-4/1.6	8	3.3–3.9	< 0.4	Dual
^58^	0.8 × 0.8	FR-4/0.8	8	2.38–2.54, 3.11–4.15	–	Dual
^20^	1.59 × 0.77	FR-4/0.8	8	3.3–5	–	Single
^59^	2.18 × 1.04	FR-4/1.2	8	4.37–5.5	–	Dual
This work	0.28 × 0.28	FR-4/1.6	12	1.7–1.9, 2.35–2.55, 3–12	< 0.28	Hexa

Thus, it can be concluded that the proposed design has packed more elements in a smaller space while maintaining a high degree of isolation between them. Further, the distinct orientation of the antenna elements offers a wider range of polarization vectors, which is highly desirable in a rich scattering and deep fading environment.

## Conclusion

In this work, a MIMO antenna that operates in the UWB, Bluetooth, and GSM bands is presented. The antenna is made up of twelve elements that are arranged in horizontal and vertical planes. The antenna diversity performance is investigated, and the values are within the limits. The proposed antenna achieves high gain and efficiency. The antenna housing effect is investigated to determine the consistency of the radiator when it is installed in a vehicle. The reflection coefficients and directivity investigated from the antenna housing effect are satisfactory. The antenna can be installed in automobiles for automotive applications such as V2V communication and ITS.
